# Treatment Outcome and Its Determinants among Patients Admitted to Stroke Unit of Jimma University Medical Center, Southwest Ethiopia

**DOI:** 10.1155/2020/8817948

**Published:** 2020-12-30

**Authors:** Ameha Zeleke Zewudie, Tolcha Regasa, Solomon Hambisa, Dejen Nureye, Yitagesu Mamo, Temesgen Aferu, Desalegn Feyissa, Tewodros Yosef

**Affiliations:** ^1^Mizan-Tepi University, College of Health Science, Department of Pharmacy, Mizan-Aman, Ethiopia; ^2^Mizan-Tepi-University, College of Medicine and Health Science, School of Public Health, Department of Epidemiology and Biostatistics, Mizan-Aman, Ethiopia

## Abstract

**Background:**

Stroke is a public health problem in Ethiopia. Despite the high prevalence of stroke in Ethiopia, there is a paucity of data with regard to drug treatment, treatment outcome, and risk factors of poor treatment outcome of stroke. Hence, this study is aimed at assessing treatment outcome and its determinants among patients admitted to stroke unit of Jimma University Medical Center (JUMC).

**Methods:**

A two-year hospital-based retrospective cross-sectional study was employed to analyze the medical records of patients admitted with stroke to stroke unit of Jimma University Medical Centre from February 1^st^, 2016 to March 30^th^, 2018. Data was entered by Epidata manager version 4.0.2 and analyzed by SPSS version 24. Multivariable logistic regression analysis with the backward stepwise approach was done to identify independent predictors of poor treatment outcome of stroke. Variables with *P* value less than 0.05 were considered as statically significant determinants of poor treatment outcome.

**Results:**

Of 220 patients with stroke admitted to the Jimma University, 67.30% were male. Nearly two thirds (63.18%) of them had poor treatment outcomes. Dyslipidimics were administered to 60% of the patients, and the most popular antiplatelet used was aspirin, which was prescribed to 67.3% the patients. Age ≥ 65 adjusted odd ratio ((AOR): 2.56; 95% CI: 1.95-9.86, *P* = 0.001), presence of comorbidity (AOR: 5.25; 95% CI: 1.08-17.69, *P* < 0.001), admission with hemorrhagic stroke (AOR: 18.99; 95% CI: 7.05-42.07, *P* < 0.001), and admission to the hospital after 24 hour of stroke onset (AOR: 4.98; 95% CI: 1.09-21.91, *P* = 0.03) were independent predictors of poor treatment outcomes.

**Conclusion:**

Substantial numbers of stroke patients had poor treatment outcomes. Elderly patients, patients diagnosed with hemorrhagic stroke, patients with comorbidity, and those with delayed hospital admission were more likely to have poor treatment outcome. Hence, frequent monitoring and care should be given for the aforementioned patients. Awareness creation on the importance of early admission should be delivered particularly for patients who have risk factors of stroke (cardiovascular diseases).

## 1. Background

According to the World Health Organization (WHO), stroke is defined as rapidly developing clinical signs of focal (or global) disturbance of cerebral function with symptoms lasting 24 hours or longer or leading to death, with no apparent cause other than of vascular origin [1]. WHO has estimated that about 15 million new stroke cases per year were diagnosed around the globe; two thirds of them were occurred in low- and middle-income countries (LMIC). In Africa, findings showed that from total of patients with stroke admitted to the hospitals, 86% of them died due to stroke [2, 3].

Stroke is among the most disabling cerebrovascular disease that result in significant amount of residual deficit leading to economic loss. This high burden of poor outcomes of stroke is not only attributable to its high in-hospital mortality but also its high physical disability and huge economic lost [4, 5]. The Global Burden of Diseases (GBD) study also showed about 80% stroke-related death that occur in LMIC indicating that developing countries carry the heavy burden of stroke-related disability, mortality, and morbidity [6, 7]. In Ethiopia, the burden and outcome of hemorrhagic and ischemic stroke vary over a time of periods and between regions [8].The majority of patients with stroke died early after admission because of stroke-related acute medical and neurological complications. Because of poor standard of care and late presentation, in-hospital death is higher, and most of the patients were discharged with severe physical disability [9, 10]. For instance, study conducted in Shashemene, Ethiopia, showed that about 45% of the patients with stroke had poor treatment outcomes [11].

The difference in the prevalence of risk factors among stroke subtypes showed that the knowledge of pathology of stroke is substantial for the management and overall care of the patients [12]. Late presentation to the hospital after the onset of stroke, underdiagnosis of hypertension (important risk factor for hemorrhagic stroke), and nonadherence to the treatments was identified as determinants of poor treatment outcome of stroke in SSA countries [9, 13]. The genetic variation also plays a significant role in the pathogenesis of stroke besides the highest burden of stroke risk factors in SSA. For instance, diabetes mellitus (DM) and hypertension were more prevalent among black than white races [14, 15]. Besides those risk factors, cardiological examination and brain imaging were infrequently performed for etiologic investigation and identification of stroke subtype in SSA because of unavailability of the equipment and lack of resource [16].

Despite high prevalence and burden of stroke in Ethiopia, there is a shortage of data regarding treatment outcome and determinants of poor treatment outcomes of stroke. To our knowledge, there is only a single study conducted in Shashemene, Ethiopia, on stroke treatment outcomes. This study used very small sample size (involved only 73 subjects) which cannot able to identify determinants of poor treatment outcomes. The shortage of date which is specific to the Ethiopian setting limits formulation of well-designed prevention strategy and treatment of stroke [9]. Because of this, SSA countries including Ethiopia have used data that came from the developed countries for prevention, follow-up, and management of stroke [17]. Hence, this study is aimed at filling these gaps through generating useful data that will guide for stroke management and determinants of poor treatment outcomes among patients with stroke admitted to stroke unit of JUMC.

## 2. Methods and Participants

### 2.1. Study Design and Setting

A two-year hospital-based retrospective cross-sectional study design was conducted to analyze the medical records of stroke patients admitted to JUMC from February 1^st^, 2016 to March 30^th^ 2018. The study was conducted in JUMC which is a tertiary hospital found in the Jimma University, Oromia region, Southwest Ethiopia, and about 346 km away from the capital city Addis Ababa, Ethiopia. Currently, the hospital provides services for approximately 20000 inpatient and 160,000 outpatient attendances yearly coming from a catchment population of over15 million.

### 2.2. Eligibility Criteria

Patients admitted to stroke unit of JUMC during the period of February 1^st^, 2016 to March 30^th^, 2018 and those who diagnosed clinically and confirmed by brain imaging as per the WHO criteria for diagnosis of stroke were included [18]. Patients under the age of 18 years, those with incomplete medical records, and patients with diagnosis of transient ischemic attack (TIA) were excluded from the study.

### 2.3. Sample Size Determination

A survey of all medical records of stroke patients hospitalized during the period of February 1^st^, 2016 to March 30^th^, 2018 was conducted to identify eligible patients for the study. Therefore, a total of 220 medical records of stroke patients who met the inclusion criteria were included in the study.

### 2.4. Data Collection Techniques and Procedure

Data was collected by trained and qualified pharmacists using checklist from the stroke unit of the hospital and supervised by one clinical pharmacy specialist. Identification card of hospitalized patients with stroke was collected from a discharge summary of the patients who had been hospitalized during a period of February 1^st^, 2016 to March 30^th^, 2018. Data collection tools were developed from the findings of studies conducted at different sites. Patients' medical records were used to collect sociodemographic characteristics, laboratory findings, treatment regimens, outcomes of treatment, and clinical data such as risk factors, subtype of stroke, clinical presentations, and vital sign of the patients. Pretest was done on 11 medical records of the patients with stroke to ensure a reliability and variability of the data collection tools.

#### 2.4.1. Outcome Measurement

The outcome variables are classified as good and poor treatment outcomes. Good treatment outcomes are defined as if the patient was discharged with improvements and/or discharged without complication, whereas poor treatment outcome is defined as if the patient was discharged with complication or referred to higher health facility or died in the hospital.

#### 2.4.2. Data Analysis

The collected data was checked, cleaned, and entered by using Epidata manager version 4.0.2.101 and analyzed by using SPSS version 24. Descriptive statics used to summarize categorical variables of sociodemographic characteristics, treatment pattern, and stroke event factors by using frequency, percent, mean, and standard deviation to describe the independent variables. Bivariate logistic regression was employed to see the association between independent variables and the outcomes of stroke. *P* value <0.25 was considered as a cutoff point for candidate selection for multivariable logistic regression analysis with the backward stepwise approach to identify independent predictors of poor treatment outcome of stroke. Odd ratio and confident interval were used to summarize the final data. Confidence interval in which 1 is not found within the interval and variable with value less than 0.05 was considered to be statically significant.

### 2.5. Standard and Operational Definition

#### 2.5.1. Stroke

As per the WHO criteria, stroke is defined as rapidly developing clinical signs that result in focal or global disturbance of the cerebral function, with symptoms lasting 24 hours or longer or leading to death with no known cause other than vascular origin [18–20].

#### 2.5.2. Ischemic Stroke

It is evidence of a recently confirmed cerebral infraction in the clinically relevant area of the brain [21, 22].

#### 2.5.3. Hemorrhagic Stroke

A type of stroke which occurs because of weakening of brain blood vessels which rapture and bleed into the surrounding tissues [23].

#### 2.5.4. Glasgow Coma Scale (GCS)

The measurement scale used to measure the level of consciousness of patients with stroke. GCS is defined as good if the patient has mild brain injury/alert, GCS (13-15) or moderate brain injury/drowsy, GCS (9-12). GCS is defined as poor if the patients had severe brain injury/unconscious, GCS (≤ 8) [11].

*Risk factors of stroke* are defined as the presence of stroke risk factors such as hypertension, smoking, DM, alcohol abuse, heart diseases, and atrial fibrillation among studied participants.

#### 2.5.5. Smoker

Smoker is defined as if the participant smoked 2 and 1 cigarette per day for male and female, respectively.

#### 2.5.6. Alcohol Abuse

Alcohol abuse is defined as a consumption of alcohol on average of ≥2 drinks and 1 drinks perday for male and female, respectively [21].

*Good treatment outcome* is defined if the patients discharged from the hospital are with improvements and without complication. *Poor treatment outcome* is defined as the patients discharged with complication or referred to higher health facility or if the patient died in the hospital [24].

## 3. Results

### 3.1. Sociodemographic Characteristics

From the total of 220 patients with stroke included in the study, nearly two thirds (67.3%) of them were male. No patient was admitted with stroke during a period in February 1^st^, 2016 to March 30^th^, 2018 with the age group of 18-24 years. Majority of the study participants were married (94.5%), and more than half (58.2%) were farmers. The mean age of the patients in this study was 62.33 ± 15.77 years. More than one third (40.4%) of studied participants were smokers while 34.54% had history of alcohol consumption (Table 1).

### 3.2. Clinical Characteristics and Risk Factors of Hospitalized Stroke Patients

Less than half of the patients (40.91%) were admitted after 24 hours of stroke onset, and none of them arrived at the hospital before 4.5 hours. The mean time of the participants from symptom onset to hospital admission was 26.45 ± 12.54 hours. The average length of hospital stay was 14 ± 3.4 days. The average time from hospital admission to initiation of antiplatelet agent and statins [for ischemic stroke] was 25 ± 6.8 hours. Greater than one third (36.82%) of the participants were improved and discharged from the hospital while 45.0% of the participants were discharged with physical disability and/or neurological deficit. The in-hospital mortality rate among the participants was 18.18%. Greater than two thirds (70.9%) of the patients were diagnosed with ischemic stroke while the remaining patients (29.1%) were diagnosed with hemorrhagic stroke. Less than one third (29.5%) of the participants complain of the left side body weakness at admission. The other common chief complaints include aphasia (23.6%), right side body weakness (21.9%), and loss of consciousness (21.4%). The most common identified risk factors of stroke were hypertension (45.9%), smoking cigarette (40.5%), and diabetic mellitus (36.4%)). More than one third (35%) of the patients developed in-hospital stroke complications. Pressure sore 40(18.2%), aspiration pneumonia (7.3%), sepsis (4.1%), seizure (3.2%), and deep venous thrombosis (DVT) (2.2%) were the identified in-hospital complications (Table 2).

### 3.3. Pharmacotherapy Pattern of Hospitalized Stroke Patients

The most commonly prescribed drugs were aspirin [ASA) plus lovastatin (41.8%) followed by aspirin plus sivastatin (18.2%). The only antiplatelet used for treatment of ischemic stroke was aspirin, which was prescribed for 67.3% of the patients. In our study, lipid-lowering therapy was prescribed for 60.0% of the participants. Among hospitalized patients with stroke, 51.4% of them were discharged with both antiplatelet and lipid-lowering drugs while 8.2% of the participants were discharged with antiplatelet only (Table 3).

Of 220 studied patients with stroke, 79.1% were prescribed with concomitant medications. Almost all of the concomitant medications were drugs used to treat cardiovascular disorders. The most commonly concomitantly used medications were enalapril 75 (43.1%), captopril 29(16.7%), unfractionated heparin (UFH) 48 (27.6), and hydrochlorothiazide 22 (12.6%) (Table 4).

### 3.4. Predictors of Poor Outcome of Stroke Patients

Variables which have a *P* value <0.25 on binary logistic regression were included in multivariable logistic regression, and analysis was done with the backward stepwise approach. Age ≥ 65 (AOR: 2.56; 95% CI: 1.95-9.86, *P* = 0.001), presence of comorbidity (AOR: 5.25; 95% CI: 1.08-17.69, *P* < 0.001), admission with hemorrhagic stroke (AOR: 18.99; 95% CI: 7.05-42.07, *P* < 0.001), and admission to the hospital after 24 hour of stroke onset (AOR: 4.98; 95% CI: 1.09-21.91, *P* = 0.03) were independent predictors of poor treatment outcomes (Table 5).

## 4. Discussion

This is the first study on stroke focusing on treatment outcome and determinants of poor treatment outcome among stroke patients in Southwest Ethiopia. In our study, 46.82% and 37.27% of the participants were found in the age group of ≥65 and 45-64 years, respectively. This result is similar with the finding reported from the Shashamane Hospital, Ethiopia [11].The possible justification is an increment of hypertension and type 2 diabetes mellitus, the two important risk factors, for cardiovascular disorder in general and stroke in particular, among patients greater than 45 years [25]. However, different from the study conducted in Black Lion Hospital, where about one third of the patients were under 34 years [26]. The mean age (SD) of the patients in our study was 62.33 ± 15.77 years. This finding is in line with the study conducted in Mekele, Ethiopia, that reported the mean age of 62.8 years [17], but higher than the finding in studies conducted at different hospitals of Ethiopia [13, 14].

In our study, stoke is more prevalent in male than female patients. This finding is similar with previous studies conducted at different health facilities [13, 24]. The possible justification might be due to high prevalence of hypertension, ischemic heart disease, peripheral artery disease, and cigarette smoking which are the major risk factors of stroke, and were higher in male than female of similar age [27]. However, the findings from some studies showed that the prevalence of stroke was higher among female [17, 28]. The possible reason might be high utilization of contraception and pregnancy-related disorders among females in the aforementioned studies.

Similar to the other studies, the finding of our study (70.9%) showed that ischemic stroke was more prevalent than hemorrhagic stroke [11, 17, 28]. In contrast to this, hemorrhagic stroke appeared to be more prevalent in studies done at different hospitals found in Adisabeba, Ethiopia [26, 29, 30]. Higher proportion of hemorrhagic stroke found in hospitals of Adisabeba was due to the fact that the prevalence of traumatic brain injury, the important risk factor of hemorrhagic stroke, secondary to car accident, was relatively higher in Adisabeba than in other parts of Ethiopia [31]. Moreover, significant numbers of hypertensive patients were not taking proper antihypertensive drugs and more likely to have uncontrolled hypertension which are an independent risk factor for hemorrhagic stroke in those studies.

With regard to the pharmacotherapy of stroke, lipid-lowering drugs were prescribed for 60.0% of patients with stroke. This finding is in line with the previous study done in Shashemene, Ethiopia [11], but lower than the finding from the study done by Abbasi et al. [32]. In our setting, the only antiplatelet prescribed for studied participants was aspirin. This is because of the limited availability of clopidogrel and unavailability of thrombolytic medications such as rTPA [13].

Unlike the previous studies conducted in Ethiopia [11, 29], the finding of the present study showed that higher percentage (63.18%) of the patients had poor treatment outcomes, of which 18.18% of patients died in the hospital while 45.00% of the patients were discharged with physical disabilities (neurological deficit/complications). The possible explanation for this discrepancy was because of higher percentage (46.82%) of elderly patients (≥65 years) which were more likely to have poor treatment outcomes in our study. In addition to this, the finding from the present study showed that the median time from stroke symptoms onset to patient arrival at the hospital was relatively longer (26.45 ± 12.54 hours) than the previous studies. Our study also indicated that unable to reach the hospital timely was the independent predictor of poor treatment outcome. Moreover, higher percentages of stroke patients with comorbidity were identified in the current study, and patients who had comorbidity were more likely to have poor treatment outcomes.

The finding of the present study showed that age ≥ 65, presence of comorbidities, admission with hemorrhagic stroke, and admission to the hospital after 24 hour of stroke onset were independent predictors of poor treatment outcomes. Elderly patients with stroke had 2.56 times more likely to have poor treatment outcomes. This finding is consistent with the findings of the studies conducted at a few facilities [33, 34]. Contrary to this, study done in Shashemene, Ethiopia, showed that advanced age did not affect the outcomes of the patients with stroke [11]. This difference might due to the fact that the previous study involved a few numbers of participants (73), which is an inadequate sample size to identify independent predictors of poor treatment outcomes in the aforementioned study.

The presences of comorbidities is also identified as an independent predictor of poor treatment outcomes in the study setting. This finding is in contrast to study done in Shashemene, Ethiopia where co-morbidity is not associated with poor treatment outcomes among patients with stroke [11]. This difference might be due to a few numbers of participants (73) which is an inadequate sample size to identify independent predictors of poor treatment outcomes in the previous study. However, our finding is in agreement with the findings reported by Fischer et al. and Sennfalt et al. which showed that comorbidity has a substantial impact on stroke treatment outcomes [35, 36]. The possible justification might be due to the impact of comorbidity conditions on the pathogenesis of inflammatory injury and subsequent recovery of stroke. The additive effect of illness burden from multiple conditions may contribute to poorer health status, higher risk of mortality, and poorer functioning through increased treatment burden. Hence, patients with comorbidities may recover more slowly and achieve lower treatment outcomes [35].

Hemorrhagic type of stroke was the other independent predictor of poor treatment outcomes. This finding is in line with the study conducted in South London where patients diagnosed with intracerebral hemorrhage had poor treatment outcomes as compared to ischemic stroke [37]. This is due to the fact that the pathophysiology of hemorrhagic stroke that may lead to spasms in the blood vessels and or increased pressure on the brain which damage brain cells in which the damaged area is unable to function properly.

We found that patients admitted after 24 hours of stroke onset were more likely to have poor treatment outcomes than patients admitted before 24 hours after the onset of stroke. In accordance to our results, previous study in North Ethiopia [38] revealed that delay admission to the hospital was the major determinant of poor treatment outcomes. The observed association of delayed admission with poor treatment outcomes in stroke patients may result poor outcomes of stroke by reducing therapeutic interventions at a time when the brain is primed for. Admission of patients within the window period has been linked with efficacy of treatment and fast recovery [39]. This finding has the following important implication. Considering the concept that “time is brain” and the majority of patients were admitted with diagnosis of ischemic stroke, these subsets of patients should have the acute treatment option with IV rTPA if available. Even though thrombolytic medications were currently not available in our country, significant delays of hospital admission may create treatment difficulties and would make such advanced treatments difficult in the future even if rTPA is available in the local hospital.

Our studies have notable limitations. First, since our study is retrospective, we were limed to use medical records. For instance, data from patients' medical records were insufficient to measure outcome variables objectively using the Modified Ranking Scale (mRS); thus, the outcome of the interest was measured subjectively. Second, we had conducted our study in a single institution of the referral hospital in Southwest Ethiopia, so that the finding of our study could not be generalized to the whole stroke patients found in Ethiopia.

## 5. Conclusion

Substantially, numbers of stroke patients have poor treatment outcomes. Aspirin and statin were the most frequently prescribed medications in management of stroke. Elderly patients, patients diagnosed with hemorrhagic stroke, patients with comorbidity, and those with delayed hospital admission were more likely to have poor treatment outcomes. Hence, frequent monitoring and care should be given for the aforementioned patients. Awareness creation on the importance of early admission should be delivered specially for patients who have risk factors of stroke (cardiovascular diseases).

## Figures and Tables

**Table 1:** Sociodemographic characteristics of patients with stroke admitted at the stroke unit of JUMC, Southwest Ethiopia, February 1^st^, 2016 to March 30^th^ 2018.

Sociodemographic characteristics	Numbers *N* = 220 (%)
Sex	
Male	148 (67.3)
Female	72 (32.7)
Age	
25-44	35 (15.9)
45-64	82 (37.27)
≥ 65	103 (46.82)
Marital status	
Single	12 (5.5)
Married	208 (94.5)
Religion	
Orthodox	52 (23.6)
Muslim	156 (70.9)
Protestant	12 (5.5)
Occupation	
Civil servant	28 (12.7)
Farmer	128 (58.2)
Unemployed	12 (5.5)
Student	8 (3.6)
House wife	44 (20.0)
Social history	
Smoker	89 (40.4)
Alcoholics	76 (34.5)
Chat	69 (31.36)
Coffee	87 (39.55)

**Table 2:** Clinical characteristics and risk factors among patients with stroke admitted at the stroke unit of JUMC, Southwest Ethiopia, February 1^st^, 2016 to March 30^th^ 2018.

Clinical characteristics	Frequency (%) *N* = 220 (%)
Patient status	
Improved and discharged	81 (36.82)
Discharged with neurologic deficit	99 (45.00)
Died in hospitals	40 (18.18)
Types of stroke	
Ischemic	156 (70.9)
Hemorrhagic	64 (29.1)
Time of admission	
4.51-12	67 (30.45)
12.01-24 0hours	63 (28.64)
> 24 hours	90 (40.91)
Length of hospital stay	
< 7days	88 (40.0)
> 7 days	132 (60.0)
Chief compliant	
Left side body weakness	65 (29.5)
Right side body weakness	48 (21.9)
Aphasia	52 (23.6)
Loss of consciousness	47 (21.4)
Failure to control both extremities	16 (7.3)
Risk factors	
Yes	27 (12.27)
No	193 (87.73)
Types of risk factors	
Hypertension	101 (45.9)
Current smokers	89 (40.5)
Diabetic mellitus	80 (36.4)
Alcohol drinkers	76 (34.5)
Heart disease	18 (8.2)
Atrial fibrillation	12 (5.5)
In-hospital stroke complication	
Yes	77 (35.00)
No	143 (65.0)
Pressure sore	40 (18.2)
Aspiration pneumonia	16 (7.3)
Sepsis	9 (4.1)
Seizure	7 (3.2)
Deep venous thrombosis (DVT)	5 (2.2)
Glasgow coma scale (GCS) during admission	
> 8	151 (68.6)
≤ 8	69 (31.4)
BMI	
< 18.5	11 (5.0)
18.5–24.9	28 (12.7)
25–29.9	99 (45.0)
30 or greater	82 (37.3)
RR during admission	
< 12 bpm	5 (2.3)
12-20 bpm	94 (42.7)
> 20 bpm	121 (55.0)
HR during admission	
< 60 bpm	45 (20.4)
61-100 bpm	78 (35.5)
> 100 bpm	97 (44.1)
BUN admission	
< 20 mg/dl	120 (54.5)
≥ 20	100 (45.5)
Scr admission	
< 1 mg/dl	113 (51.4)
≥ 1 mg/dl	107 (48.6)
Total cholesterol	
< 200 mg/dl	132 (60.0)
≥ 200 mg/dl	88 (40.0)
HDL during admission	
≥ 40 mg/dl	166 (75.5)
< 40 mg/dl	54 (24.5))
LDL admission	
< 100 mg/dl	143 (65.0)
≥ 100 mg/dl	77 (35.0)
Triglycerides during admission	
> 150 mg/dl	122 (55.5)
≤ 150 mg/dl	98 (44.5)

**Table 3:** Drugs used for treatment of stroke among patients admitted at the stroke unit of JUMC, Southwest Ethiopia, February 1^st^, 2016 to March 30^th^ 2018.

Pharmacotherapy pattern for stroke patients		Number *N* = 220 (%)
In-hospital drugs used for treatment of stroke	Patients with no drugs for strokes	72 (32.7)
	ASA 81 mg only	16 (7.3)
	ASA 81 mg and lovastatin 40 mg combination	92 (41.8)
	ASA 81 mg and simvastatin 40 mg combination	40 (18.2)
Stroke drugs prescribed on hospital discharge	ASA 81 mg	18 (8.2)
	ASA 81 mg and lovastatin 40 mg combination	73 (33.2)
	ASA 81 mg and simvastatin 40 mg combination	40 (18.2)
	Patients discharged without drugs for strokes	15 (6.8)

**Table 4:** Concomitantly used drugs among patients with stroke admitted at the stroke unit of JUMC, Southwest Ethiopia, February 1^st^, 2016 to March 30^th^ 2018.

Class of drugs	Type of drugs	Number *N* = 174 (%)
ACE inhibitors	Enalapril	75 (43.1)
	Captopril	29 (16.7)
Beta blockers	Atenolol	7 (4.0)
	Metoprolol	3 (1.7)
	Propranolol	5 (2.9)
Calcium channel blockers	Amlodipine	14 (8.0)
	Nifedipine	7 (5.0)
Diuretics	Furosemide	4 (2.3)
	Hydrochlorothiazide	22 (12.6)
Anticoagulant drugs	Warfarin	5 (2.9)
	UFH	48 (27.6)
Antiepileptic drugs	Phenytion	13 (7.5)
Other	Mannitol	25 (14.4)

**Table 5:** Predictors of poor treatment outcomes among patients with stroke admitted at the stroke unit of JUMC, Southwest Ethiopia, February 1^st^, 2016 to March 30^th^ 2018.

Variables	Good outcome *N* (%)	Poor outcome *N* (%)	AOR (95% CI)	*P* value
Age				
25-44	17 (7.73)	18 (8.18)	1	
45-64	40 (18.18)	42 (19.09)	1.02 (0.09-4.98)	0.98
≥ 65	24 (10.91)	79 (35.91)	2.56 (1.95-9.86)	0.001
Presence of comorbidities				
No	21 (9.55)	6 (2.73)	1	
Yes	60 (27.27)	133 (60.45)	5.25 (1.08-17.69)	<0.001
Types of stroke				
Ischemic	74 (33.64)	82 (37.27)	1	
Hemorrhagic	7 (3.18)	57 (25.91)	18.99 (7.05-42.07)	<0.001
Time of admission				
< 12hour	30 (13.64)	37 (16.82)	1	
12-24hour	27 (12.27)	36 (16.36)	1.01 (0.12-5.13)	0.71
> 24hour	24 (10.91)	66 (30.00)	4.98 (1.09-21.91)	0.03

## Data Availability

The data used to support the findings of this study are included within the article.

## Abbreviations

AOR: Adjusted odd ratio
ASA: Aspirin
DM: Diabetes mellitus
GBD: The Global Burden of Disease
GCS: Glasgow Coma Scale
HS: Hemorrhagic stroke
JUMC: Jimma University Medical Center
LMIC: Low middle-income countries
SSA: Higher in sub-Saharan countries
TIA: Transient ischemic attack
UFH: Unfractionated heparin
WHO: World Health Organization.

## References

[1] WHO MONICA Project Principal Investigators (2011). The world health organization MONICA project (monitoring trends and determinants in cardiovascular disease): a major international collaboration. *Journal of Clinical Epidemiology*.

[2] Ezejimofor M. C., Uthman O. A., Maduka O. (2017). Stroke survivors in Nigeria: A door-to-door prevalence survey from the Niger Delta region. *Journal of the Neurological Sciences*.

[3] Jauch E. C., Saver J. L., Adams H. P. (2013). Guidelines for the early management of patients with acute ischemic stroke: a guideline for healthcare professionals from the American Heart Association/American Stroke Association. *Stroke*.

[4] Divyant R., Amit V. (2016). The study of clinical profile and risk factor in tertiary care hospital. *International Journal of Scientific Research*.

[5] Chidiogo O., Chukwuemeka N., Onwuchekwa R., Chinwe R. O., Deborah O., Eweputanna L. I. (2015). Computerized tomography and clinical correlation of stroke diagnosis in University of Port Harcourt Teaching Hospital. *Journal of Medicine and Medical Sciences*.

[6] Mukherjee D., Patil C. G. (2011). Epidemiology and the global burden of stroke. *World Neurosurgery*.

[7] Ghandehari K. (2011). Barriers of thrombolysis therapy in developing countries. *Stroke research and Treatment*.

[8] Krishnamurthi R. V., Feigin V. L., Forouzanfar M. H. (2013). Global and regional burden of first-ever ischaemic and haemorrhagic stroke during 1990-2010: findings from the global burden of disease study 2010. *The Lancet Global Health*.

[9] Grefe E. S., Mitiku T., Getahun S. (2015). Risk factors, clinical pattern and outcome of stroke in a referral hospital, Northwest Ethiopia. *Clinical Medicine Research*.

[10] Deresse B., Shaweno D. (2015). Epidemiology and in-hospital outcome of stroke in South Ethiopia. *Journal of the Neurological Sciences*.

[11] Tegegne G. T., Berhanu T., Peter N. (2018). Treatment outcomes and associated factors among hospitalized stroke patients at Shashemene Referral Hospital, Ethiopia. *Stroke Research and Treatment*.

[12] Moran A., Forouzanfar M., Sampson U., Chugh S., Feigin V., Mensah G. (2013). The epidemiology of cardiovascular diseases in sub-Saharan Africa: the global burden of diseases, injuries and risk factors 2010 Study. *Progress in Cardiovascular Diseases*.

[13] Fekadu G., Wakassa H., Tekle F. (2019). Stroke event factors among adult patients admitted to stroke unit of Jimma University Medical Center: prospective observational study. *Stroke Research and Treatment*.

[14] Owolabi M. O., Agunloye A. M. (2013). Which risk factors are more associated with ischemic rather than hemorrhagic stroke in black Africans?. *Clinical Neurology and Neurosurgery*.

[15] Fekadu G., Chelkeba L., Melaku T., Tegene E. (2018). Pathological sub types and diagnostic protocols of stroke among adult patients admitted to Jimma University medical center, south West Ethiopia. *Journal of Neurology & Neurophysiology*.

[16] Ekeh B., Ogunniyi A., Isamade E., Ekrikpo U. (2015). Stroke mortality and its predictors in a Nigerian teaching hospital. *African Health Sciences*.

[17] Al-Hashel J. Y., Al-Sabah A.-A., Ahmed S. F. (2016). Risk factors, subtypes, and outcome of ischemic stroke in Kuwait: a National Study. *Journal of Stroke and Cerebrovascular Diseases*.

[18] Jowi J. O., Mativo P. M. (2008). Pathological sub-types, risk factors and outcome of stroke at the Nairobi hospital, Kenya. *East African Medical Journal*.

[19] Nkoke C., Lekoubou A., Balti E., Kengne A. P. (2015). Stroke mortality and its determinants in a resource-limited setting: a prospective cohort study in Yaounde, Cameroon. *Journal of the Neurological Sciences*.

[20] Wu C. Y., Wu H. M., Lee J. D., Weng H. H. (2010). Stroke risk factors and subtypes in different age groups: a hospital-based study. *Neurology India*.

[21] Dash D., Bhashin A., Pandit A. K. (2014). Risk factors and etiologies of ischemic strokes in young patients: a tertiary hospital study in North India. *Journal of Stroke*.

[22] Kuriakose C., Shifafya M. N., Tarakan N. S., Sattanathan K., Kumar R. S. (2016). A prospective study of clinical profile of stroke in a tertiary care hospital. *Asian Journal of Pharmaceutical and Clinical Research*.

[23] Sarkar D., Halder S., Saha B., Biswas P. (2016). A study of stroke patients with respect to their clinical and demographic profile and outcome. *International Journal of Research in Medical Sciences*.

[24] Patne S. V., Chintale K. N. (2016). Study of clinical profile of stroke patients in rural tertiary health care Centre. *International Journal of Advances in Medicine*.

[25] Murakami K., Asayama K., Satoh M. (2017). Risk factors for stroke among young-old and old-old community-dwelling adults in Japan: the Ohasama study. *Journal of atherosclerosis and thrombosis*.

[26] Zenebe G., Alemayehu M., Asmera J. (2005). Characteristics and outcomes of stroke at TikurAnbessa Teaching Hospital, Ethiopia. *Ethiopian Medical Journal*.

[27] Appelros P., Stegmayr B., Terént A. (2009). Sex differences in stroke epidemiology: a systematic review. *Stroke*.

[28] Alkali N. H., Ayeni O. A., Bwala S. A., Osi-Ogbu O., Akano A. O., Alabi P. (2013). Stroke risk factors, subtypes and 30 day case fatality in Abuja, Nigeria. *Nigerian Medical Journal*.

[29] Bikila G., Takele M., Tesfaye B., Abera A. (2017). Assessment of risk factors and treatment outcome of stroke admissions at St. Paul’s Teaching Hospital, Addis Ababa, Ethiopia. *Journal of Neurology & Neurophysiology*.

[30] Asefa G., Mesere S. (2010). CT and clinical correlation of stroke diagnosis, pattern and clinical outcome among stroke patients visting Tikur Anbessa hospital. *Ethiopian Medical Journal*.

[31] Abegaz T., Gebremedin S. (2019). Magnitude of road traffic accident related injuries and fatalities in Ethiopia. *PLoS One*.

[32] Abbasi M., Ali M. (2012). Prescribing pattern of drugs in stroke patients: a prospective study. *Archives of Pharmacy Practice*.

[33] Mulat B., Mohammed J., Yeseni M., Alamirew M., Dermello M., Asemahagn M. A. (2016). Magnitude of stroke and associated factors among patients who attended the medical ward of FelegeHiwot Referral Hospital, Bahir Dar town, Northwest Ethiopia. *Ethiopian Journal of Health Development*.

[34] Nedeltchev K., Renz N., Karameshev A. (2010). Predictors of early mortality after acute ischaemic stroke. *Swiss Medical Weekly*.

[35] Fischer U., Arnold M., Nedeltchev K. (2006). Impact of co-morbidity on ischemic stroke outcome. *Acta Neurologica Scandinavica*.

[36] Sennfält S., Pihlsgård M., Petersson J., Norrving B., Ullberg T. (2020). Long-term outcome after ischemic stroke in relation to co-morbidity–an: observational study from the Swedish stroke register (Riksstroke). *European Stroke Journal*.

[37] Bhalla A., Wang Y., Rudd A., Wolfe C. D. (2013). Differences in outcome and predictors between ischemic and intra-cerebral hemorrhage: the South London Stroke Register. *Stroke*.

[38] Sennay A. G., Yang S. Y. (2016). Types, risk profiles, and outcomes of stroke patients in a tertiary teaching hospital in northern Ethiopia. *ENeurologicalSci*.

[39] Nayak A. R., Husain A. A., Lande N. H. (2015). Impact of admission time on treatment and outcome of stroke in patients admitted to tertiary care hospital: a pilot study from Central India. *Journal of Clinical and Diagnostic Research*.

